# Spatio-temporal evolution and influencing mechanism of the COVID-19 epidemic in Shandong province, China

**DOI:** 10.1038/s41598-021-86188-0

**Published:** 2021-04-09

**Authors:** Jinlong Shi, Xing Gao, Shuyan Xue, Fengqing Li, Qifan Nie, Yangfan Lv, Jiaobei Wang, Tingting Xu, Guoxu Du, Gang Li

**Affiliations:** 1 Department of Physical Education, Northwest University, Xi’an, 710127 China; 2 College of Urban and Environmental Sciences, Northwest University, Xi’an, 710127 China; 3 College of Culture and Heritage, Northwest University, Xi’an, 710127 China; 4 Alabama Transportation Institute, The University of Alabama, Tuscaloosa, AL 35487 USA; 5 Shaanxi Key Laboratory of Earth Surface System and Environmental Carrying Capacity, Xi’an, 710127 China

**Keywords:** Diseases, Health care

## Abstract

The novel coronavirus pneumonia (COVID-19) outbreak that emerged in late 2019 has posed a severe threat to human health and social and economic development, and thus has become a major public health crisis affecting the world. The spread of COVID-19 in population and regions is a typical geographical process, which is worth discussing from the geographical perspective. This paper focuses on Shandong province, which has a high incidence, though the first Chinese confirmed case was reported from Hubei province. Based on the data of reported confirmed cases and the detailed information of cases collected manually, we used text analysis, mathematical statistics and spatial analysis to reveal the demographic characteristics of confirmed cases and the spatio-temporal evolution process of the epidemic, and to explore the comprehensive mechanism of epidemic evolution and prevention and control. The results show that: (1) the incidence rate of COVID-19 in Shandong is 0.76/100,000. The majority of confirmed cases are old and middle-aged people who are infected by the intra-province diffusion, followed by young and middle-aged people who are infected outside the province. (2) Up to February 5, the number of daily confirmed cases shows a trend of “rapid increase before slowing down”, among which, the changes of age and gender are closely related to population migration, epidemic characteristics and intervention measures. (3) Affected by the regional economy and population, the spatial distribution of the confirmed cases is obviously unbalanced, with the cluster pattern of “high–low” and “low–high”. (4) The evolution of the migration pattern, affected by the geographical location of Wuhan and Chinese traditional culture, is dominated by “cross-provincial” and “intra-provincial” direct flow, and generally shows the trend of “southwest → northeast”. Finally, combined with the targeted countermeasures of “source-flow-sink”, the comprehensive mechanism of COVID-19 epidemic evolution and prevention and control in Shandong is revealed. External and internal prevention and control measures are also figured out.

## Introduction

As of July 29, 2020, 84,165 confirmed COVID-19 cases and 4634 deaths were reported in China. At present, the domestic epidemic prevention and control work has achieved remarkable results, and this cycle of the epidemic peak has passed^1^, although the pandemic overseas is spreading, and the global prevention and control situation is still stark.

After the outbreak of the epidemic, scientists quickly carried out research from various perspectives. In the field of virology and biomedicine, the host of SARS-CoV-2 was bat through genome comparison and recombinant genome sequence^2,3^. In the field of epidemiology, models are often used to predict the development of epidemic in different areas. For example, Li et al.^4^ compared the *R*_0_ value under SIR model with the actual value, emphasizing that strict control measures are essential to prevent the spread of the epidemic; Bai et al.^5^ found that the *R*_0_ value of COVID-19 in Shaanxi province is about 2.95, far higher than that of SARS and MERS. In the field of pharmacy, focus is on the treatment of traditional Chinese medicine and Western medicine, syndrome differentiation and classification, and a reasonable medication plan has been put forward. For example, researchers analyzed the clinical and pharmacological basis of Chinese and Western medicine for COVID-19 and the treatment plan is being optimized^6–10^ analyzed retrospectively the clinical data of 52 patients with new coronavirus, and pointed out that the combination of traditional Chinese medicine and Western medicine had a significant effect on the treatment of this disease. In the field of medicine, Li et al.^11^ predicted the potential transmission mode by analyzing the clinical characteristics of patients with special symptoms. The elderly male population with a history of disease was more susceptible to infection by revealing the demographic, clinical and radiologic characteristics of the confirmed cases^12,13^.

Geographical spatial analysis is focused on the relationship between human and environment and the evolution of spatio-temporal pattern, which will play an important role in the study of epidemic spread at the individual and broad masses levels. For example, Jin et al.^14^ revealed the spatio-temporal evolution of COVID-19 in Shenzhen, China, put forward an accurate prevention and control measure system of “time–space–human” mutual feedback and integration; Wang et al.^9^ found the spatial flow of the epidemic to Shaanxi province had the particularity of a single occurrence core of confirmed cases and significant differentiation in space. In view of this, this paper takes Shandong province, a region with a high incidence of epidemic cases though about 900 km away from Hubei province, as the study area, to reveal the sociodemographic characteristics of confirmed cases, the spatiotemporal evolution process of the epidemic, and explore the integrated mechanism of epidemic evolution and prevention and control in Shandong, so as to provide a reference for in-depth research and scientific prevention and control.

## Materials and methods

### Study area

**Figure 1:**
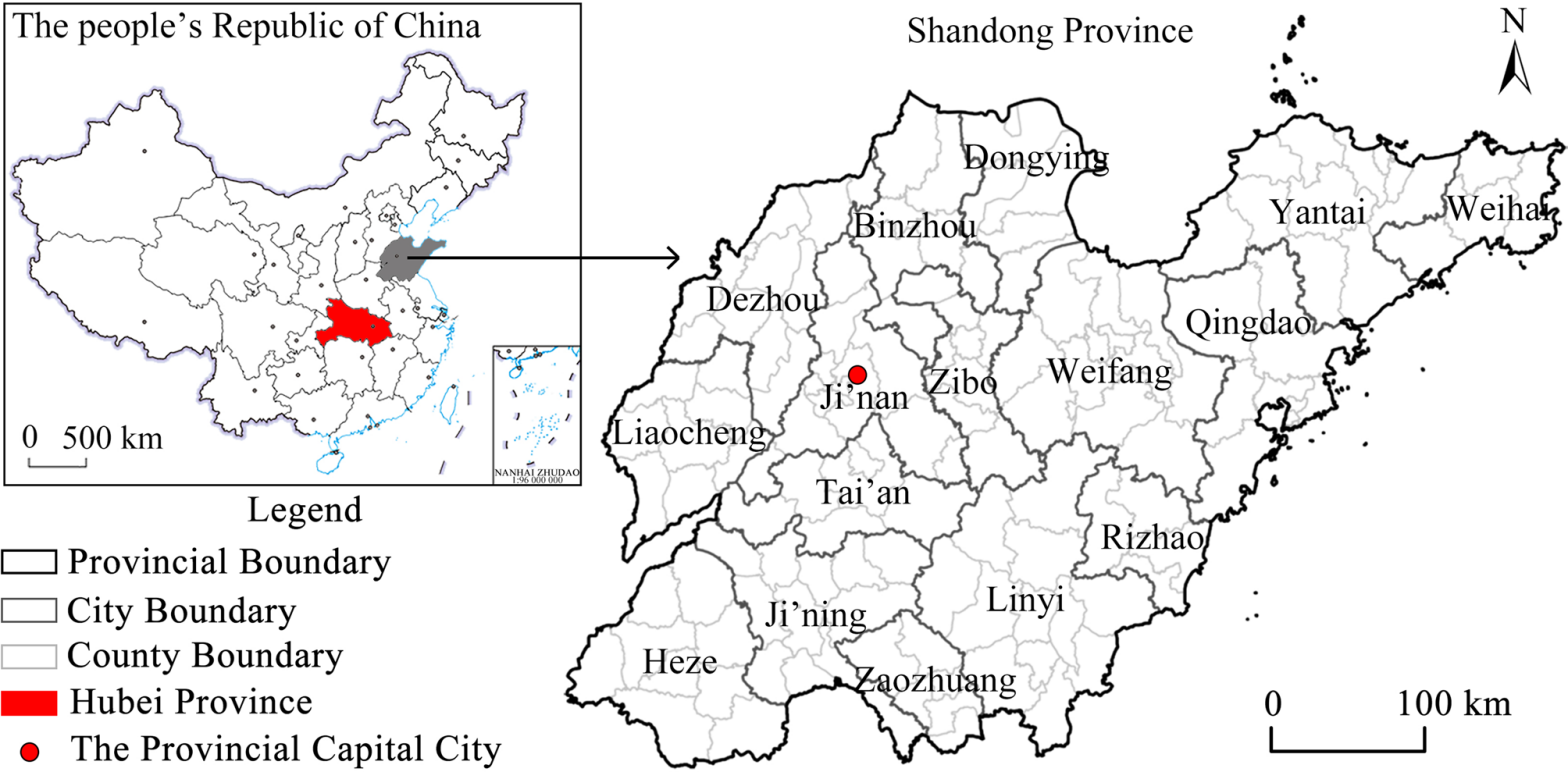
Location map of the study area (this graph is produced by using ArcGIS 10.3. http://www.esri.com; the base map is from China Standard Map Service. http://bzdt.ch.mnr.gov.cn/).

Shandong province is located in the east coast of China, bordering Hebei, Henan, Anhui and Jiangsu and facing South Korea across the sea (Fig. 1). It has jurisdiction over 16 provincial-level cities, 139 counties, county-level cities and municipal districts. As a province with a large population, Shandong was seriously affected by the epidemic. As of March 12, 2020, the province reported 759 confirmed COVID-19 cases, 1 imported case and 7 deaths, which constitutes a typical case area for the study of the spatio-temporal evolution of the region with high epidemic incidence albeit far away from the epidemic epicenter in China.

### Research data

The research data include: (1) epidemic data are based on the daily updates from all levels of Health Commissions across Shandong, and cases revealed by official media (National Health Commission of the People’s Republic of China: http://www.nhc.gov.cn/xcs/xxgzbd/gzbd_index.shtml; Health Commission of Shandong Province: http://wsjkw.shandong.gov.cn/ztzl/rdzt/qlzhxxgzbdfyyqfkgz/index.html). The data were described in Chinese, including details of case number, genders, age, signs and symptoms, date of diagnosis, relations with other cases, and time–space information. At the moment, only a few cases are missing. In the case 009 in Zibo, patient’s age is missing, in case 002 in Ji’ning, official details are missing and in the 207 cases in Rencheng Prison, official information is missing (Epidemic Data from Ji’nan Municipal Health Commission: http://jnmhc.jinan.gov.cn/col/col50360/index.html). The data were further filed by human collection and interpretation. In total, 760 cases were collected, among them, 552 showed clear gender information, 551 showed age, while 215 displayed symptom information. (2) the population and economic data came from the 2019 Statistical Yearbook of Shandong Provincial Bureau of Statistics (http://tjj.shandong.gov.cn/tjnj/nj2020/indexch.htm). (3) spatial data, and administrative boundary were from 1:1 million vector data provided by the National Basic Geographic Information Center (http://www.ngcc.cn/).

### Methods

#### Text analysis

Text analysis refers to quantifying information by extracting the expression and features of language, text, image and other carriers. In this paper, the age, gender, occupation, time–space migration path and other information were obtained by manual interpreting the texts of confirmed cases.

#### Spatial autocorrelation analysis (Moran’s *I*)

Spatial autocorrelation refers to the correlation degree of the same variable in different spatial positions, which represents the spatial aggregation degree of the spatial unit. In this paper, the global spatial autocorrelation Moran’s *I* index is introduced to reflect the clustering degree of the spatial distribution of confirmed cases in Shandong province. The formula for calculation is as follows:

1 $${\mathrm{Moran}}{\text{'}}I=\frac{n\sum_{i=1}^{n}\sum_{j=1}^{n}{w}_{ij}({x}_{i}-{\bar{x}})({x}_{j}-{\bar{x}})}{\sum_{i=1}^{n}\sum_{j=1}^{n}{w}_{ij}\sum_{i=1}^{n}{({x}_{i}-\bar{x})}^{2}}.$$

In the equation above, *I* is the global Moran index. *x*_*i*_ and *x*_*j*_ are the numbers of confirmed cases in provinces *i* and *j*. $\bar{x}$ is the average value of the confirmed cases, and *w*_*ij*_ is the spatial relationship between provinces *i* and *j* (adjacent relationship: 1, otherwise: 0).

#### Standard deviational ellipse analysis

Standard deviational ellipse (SDE) is a statistical method to represent the spatial distribution characteristics of geographical features^15^. The long half axis represents the distribution direction of geographical elements, while the short half axis represents the distribution range. The greater the difference between the two, the more obvious the spatial directionality of geographical elements are. In this paper, standard deviational ellipse is used to reveal the spatial distribution trend of confirmed cases. The formula for calculation is as follows:

2 $${\mathrm{SDE}}_{\mathrm{x}}=\sqrt{\frac{\sum_{\mathrm{i}=1}^{\mathrm{n}}{({\mathrm{x}}_{\mathrm{i}}-\bar{\mathrm{x}})}^{2}}{\mathrm{n}}}$$

3 $${\mathrm{SDE}}_{\mathrm{y}}=\sqrt{\frac{\sum_{\mathrm{i}=1}^{\mathrm{n}}{({\mathrm{y}}_{\mathrm{i}}-\bar{\mathrm{y}})}^{2}}{\mathrm{n}}.}$$

In the equation above, *x*_*i*_, *y*_*i*_ represent the geographic coordinates of the confirmed case *I*. ($\bar{x}$, $\bar{y}$) represent the average center of the confirmed case, and n is the total number of confirmed cases.

### Consent for publication

All the co-authors consent the publication of this work.

## Sociodemographic characteristics of confirmed COVID-19 cases in Shandong province

### Demographic characteristics

#### Incidence rate

As of March 12, 2020, 760 cases (incidence rate 0.76/100,000) were diagnosed in Shandong province, 739 cases were cured, 7 cases died, and 1 serious case is still alive. The epidemic affected 15 provincial-level cities, 98 counties, county-level cities and municipal districts. In addition, 17,325 close contacts (close contact rate 17.24/100,000) were reported, and the average number of persons each confirmed case was in close contact with was 23. Except for 16,970 suspected and confirmed cases including those released from medical observation, 355 cases were undergoing medical observation (0.35/100,000). So far, Shandong has been seriously affected by the epidemic with the cumulative number of confirmed cases ranking the eighth in China.

#### Gender and age structure

**Figure 2:**
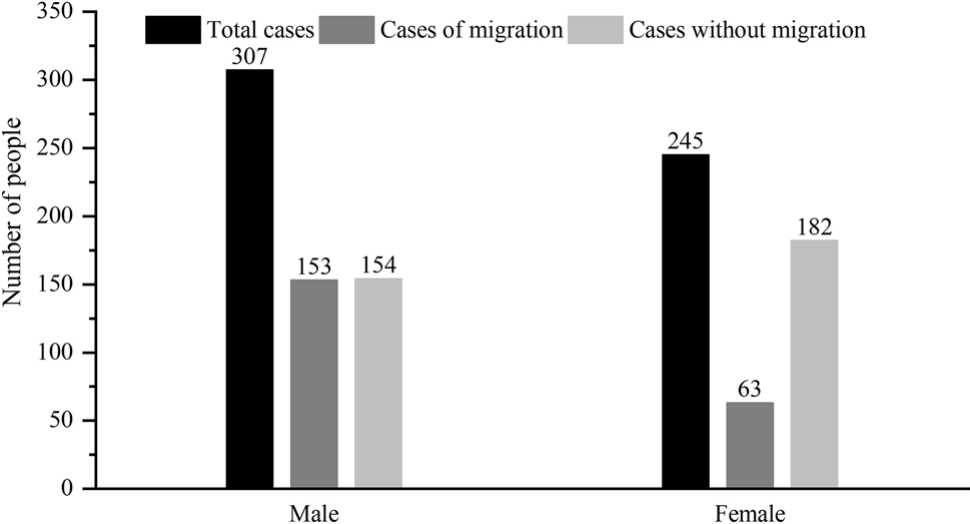
Gender characteristics of confirmed COVID-19 cases in Shandong province.

307 confirmed cases (55.62%) were male and 245 (44.38%) were female, with the former being slightly more than the latter (Fig. 2). Among the 552 cases with explicit age, the age distribution is generally characterized by “high in the middle and low in both ends”, of which, 412 cases (74.77%) are mainly aged between 30 and 66, and 139 cases under 30 and over 66. The youngest confirmed case is 9 months, while the oldest is 91, and the age span is large.

**Figure 3:**
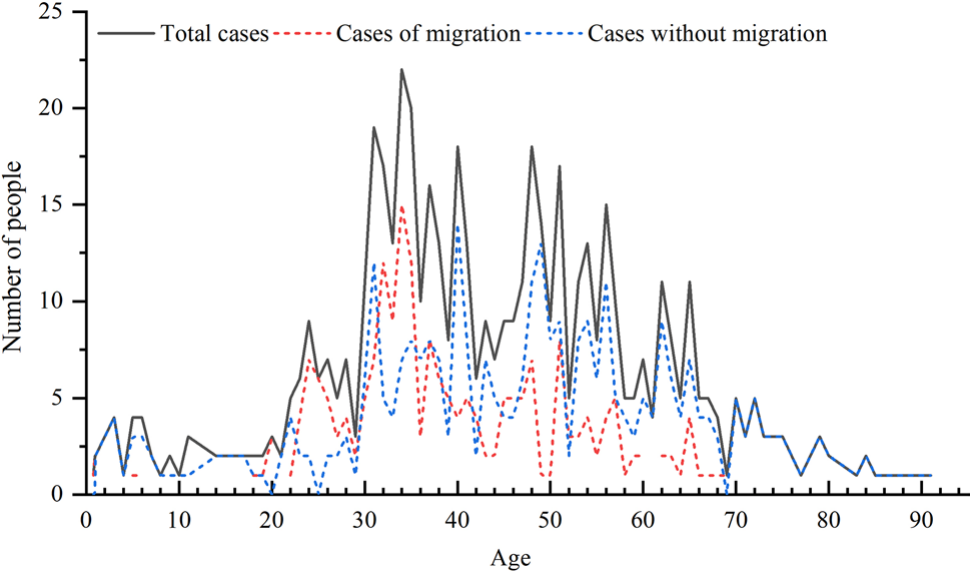
Age distribution characteristics of confirmed COVID-19 cases in Shandong province.

From the perspective of mobility of confirmed cases: among the 216 confirmed cases who traveled across provinces and cities, 153 were male and 63 were female, with a ratio of 2.43:1 and the number of male was much higher than that of female. In terms of age, the age change of confirmed cases caused by migration was consistent with the change of the total number of confirmed cases, but the span was relatively small and concentrated in 24–57 years old, mainly in middle-aged and young people without cases of migrated people over 70 years old (Fig. 3). The reason is that young and middle-aged men have a wide range of activities and mobility, and their chance to contact the virus is greater than that of women, so they become the main communicators of the epidemic in Shandong. Among the 336 confirmed cases who have never travelled across provinces and cities, 154 were male, and 182 were female, with the number of women being slightly greater than that of male. In terms of age, the overall age change was consistent with the total number, and 65.78% of the confirmed cases were concentrated over 40 years old, indicating that the middle-aged and the elderly were the main group of internal infection, and the gender was basically balanced.

The discovery resembled the cases that appeared in Spain^16^, Columbia^17^, New Zealand^18^, India^19^ yet contrasted with that of Pernambuco, Brazil^20^ and Canada^21^. The discovery further indicated that gender would not cause any difference when patients were infected by COVID-19. The larger proportion of male patients in Shandong was due to the fact that more men were located outside the province for living, working and study. As the criteria of social structure, social roles and epidemic prevention vary, the situations among different countries and regions differ. Age-wise, the patients infected appeared high in the middle age while low at both young and old age. In other words, there was a large age range, with young adults and middle-aged people being the main target. Due to the fact that COVID-19 spreads by contact and respiratory droplets, these two groups are more likely to be infected due to their close social contact and their mobility. Therefore, it is necessary to enforce the surveillance of young adults and middle-aged in addition to an epidemic prevention measure which covers the whole population.

### Infection routes

**Figure 4:**
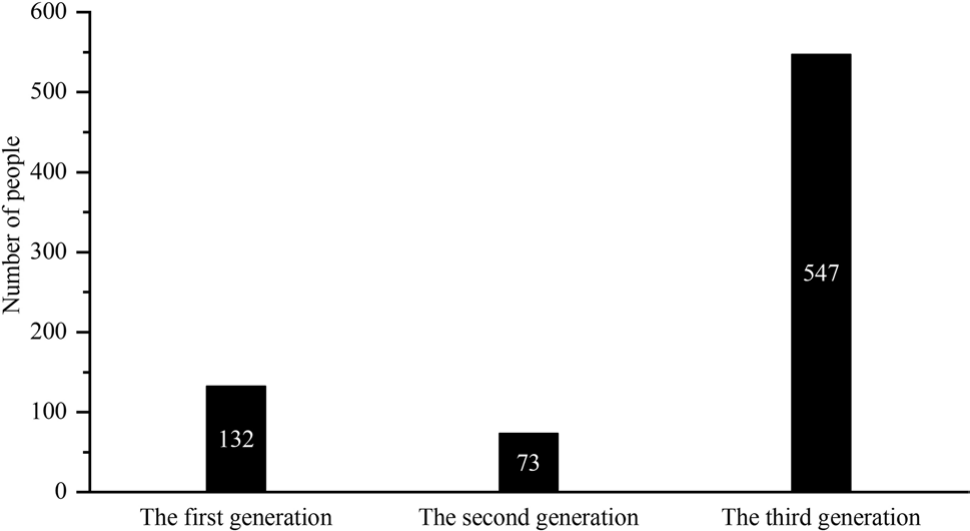
Infection generation of confirmed COVID-19 cases in Shandong province.

There are three conditions for the spread of epidemic, i.e. the source of infection, the route of transmission and the susceptibility of the population. Based on the characteristics of this epidemic and the spatio-temporal migration of confirmed cases, we analyzed the route of transmission. At present, since there is no detailed report on the infection process and activity track of confirmed cases in Wuhan or even Hubei, Hubei in this paper is regarded as the “epidemic black box” of the epidemic of China. The confirmed cases in the study area were divided into three infection generations by artificial interpretation of spatiotemporal trajectory and infection experience: the confirmed cases living and working in Hubei for a long time were defined as the first generation of infected persons (imported), the confirmed cases directly contacting the first generation of infected persons or going to Hubei for a short time before and after the epidemic were defined as the second generation of infected persons (mixed transitional), and the confirmed cases that had no sojourn in Hubei or exposure history and cannot be identified as the second generation of infection are defined as the third generations of infection or above (diffuse). According to this statistics of the infection generations of confirmed cases in Shandong (except for 8 cases without official information) (Fig. 4). the result showed that 71.97% of the confirmed cases in Shandong were internal spread type, followed by imported type and transitional type. There are 132 cases of imported infections, who are returnees after working, going to school or living in Wuhan and other areas of Hubei province for a long time; 73 cases of transitional infection, which are mainly those who had close contact with the returnees from Hubei province and have visited family members, toured or made business trips to Hubei; 547 cases of internal spread infection, who are local residents without traveling outside Shandong but had close contact with the above two types of cases.

### Types of spread

According to the information of confirmed cases, the spread types are divided into 8 categories (Table 1), namely, staying in other regions (274), major events (207), family gathering (148), visiting family members (32), travel (28), work and study outside the province (55), overseas contact (1) and public places (26).

**Table 1:** Infection type and social relations of confirmed COVID-19 cases in Shandong province.

Infection type	Social relations	Number
Staying in other regions	Long-term or short-term residence	274
Major events	Ren Cheng prison	207
Family gathering	Father–son relationship, mother–son relationship, married couples relationship, mother-in-law relationship, mother–daughter relationship, other kinship	148
Work and study outside the province	Work, study	55
Visiting family members	Inter-provincial, province-wide	32
Travel	International travel, domestic travel	28
Overseas contact	From Italy	1
Public places	Market, supermarket, hospital	26

Close contact refers to infection without clear social relationship.

**Figure 5:**
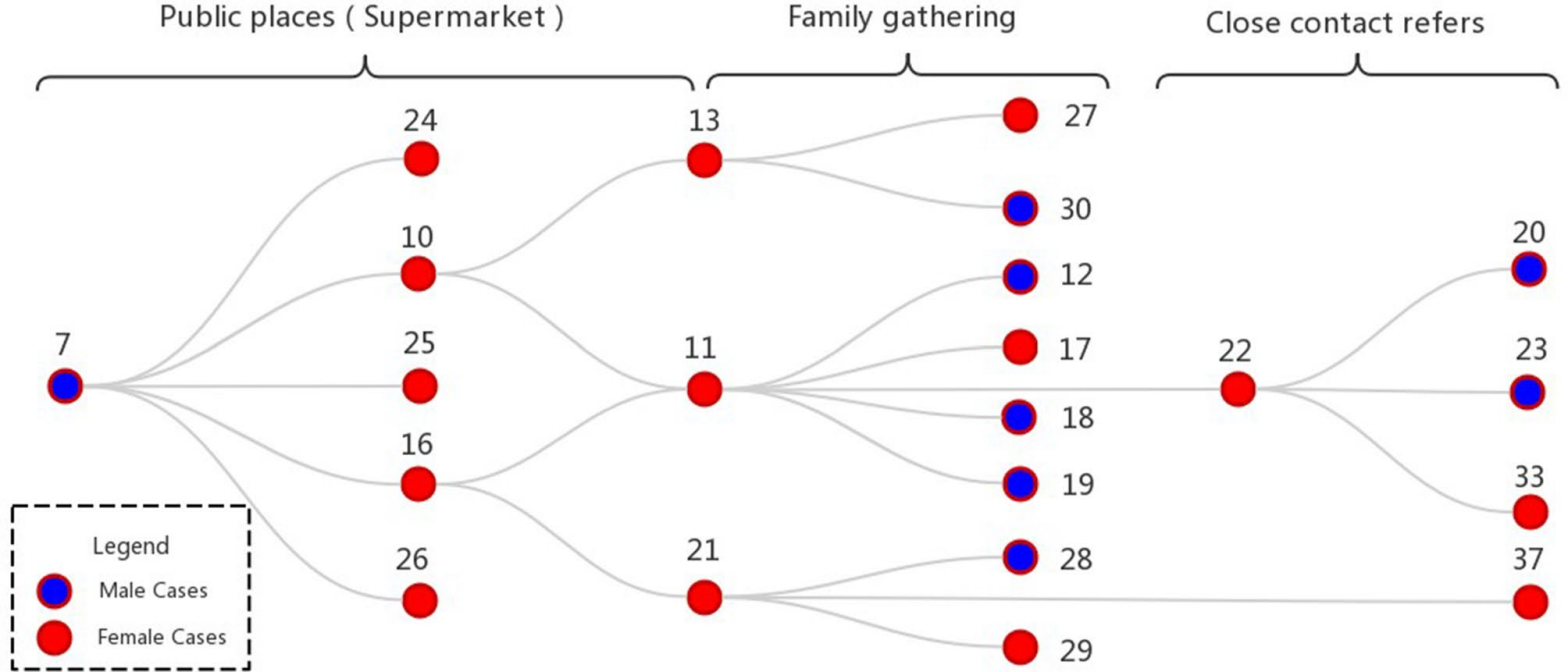
The epidemic spreading network of a typical COVID-19 super spreader in Liaocheng City. *Note* Close contact refers to infection without clear social relationship.

According to the transmission map of confirmed cases in Shandong, apart from the “Rencheng prison incident”, the largest number of people were directly or indirectly infected by a man surnamed Nie in Liaocheng. Based on this, we analyzed the social network relationship of the spread of epidemic (Table 1), and drew a typical transmission map of super infectors (Fig. 5). It can be seen from the map that there are three types of spread, namely, public place, family gathering and close contact. Among them, there are 8 confirmed male cases and 14 female cases. In public places, supermarkets are the main places of spread, with 9 cases of infection. Among them, cases 10, 11, 13, 21, 26 are colleagues, while cases 16, 22, 24, 25 are supermarket promoters. There are 8 cases involving family gathering, including 2 groups of married couples (cases 11 and 12, cases 13 and 30), 3 groups of father–son relationship (cases 11 and 18, cases 11 and 19, cases 21 and 28), 2 groups mother–son relationship (cases 11 and 17, cases 21 and 29), 1 group of mother–daughter relationship (case 13 and 27). There were 4 close contacts, of which case 20 and case 33 were infected by close contact with case 22 on the way to the supermarket, cases 23, 37, 21 and 22 were infected by close contact for unknown reasons. It was speculated that they might be neighbors and friends.

### Profiling susceptible and high-risk groups

**Figure 6:**
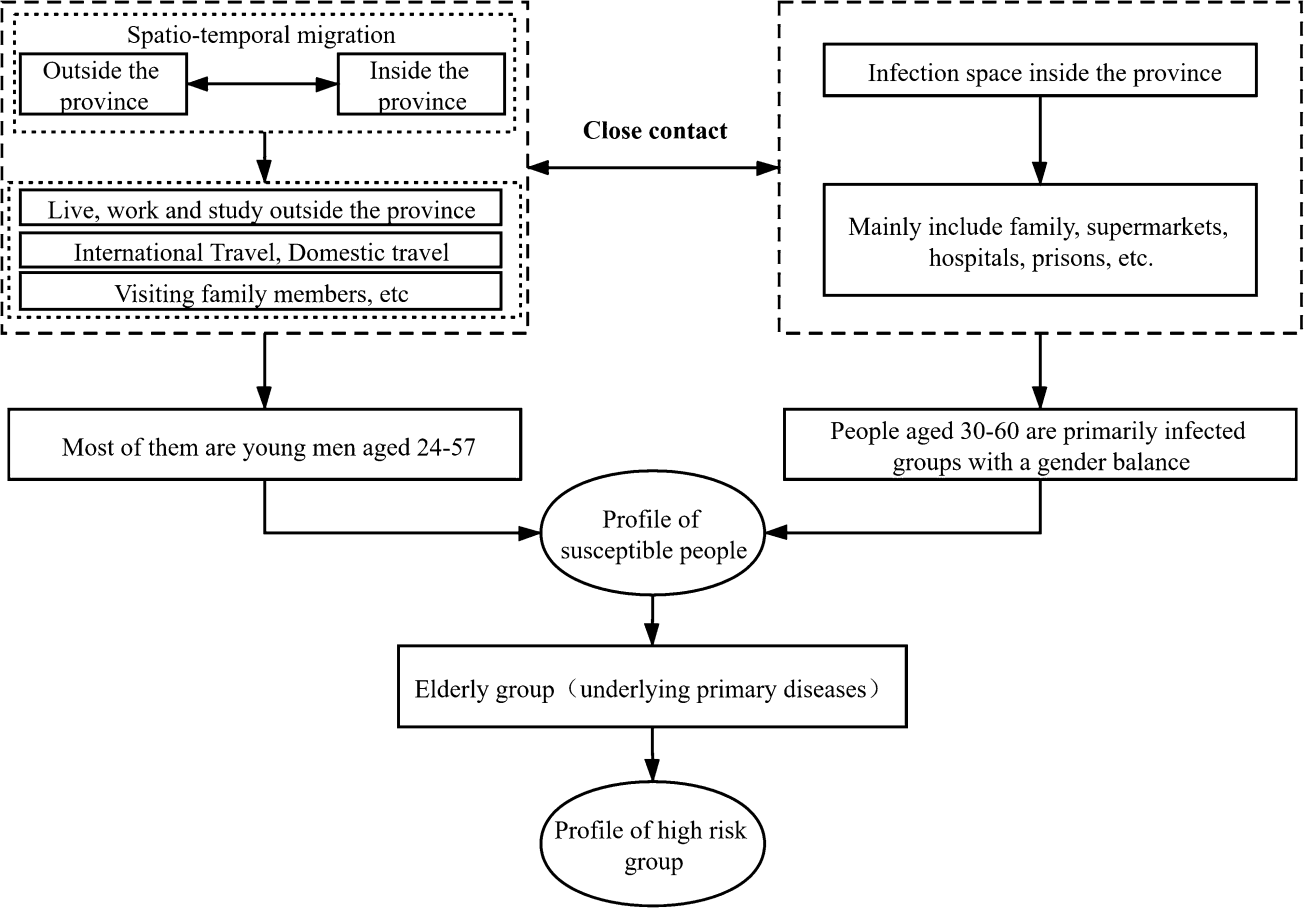
Profiling susceptible and high-risk groups of COVID-19 in Shandong province.

Combining the epidemiological characteristics and spread type features of confirmed cases in Shandong, a comprehensive profile of susceptible and high-risk groups was made (Fig. 6). There are two main types of susceptible groups: first, group travelling across provinces and cities, mainly returnees who have lived, traveled, visited and worked outside the province, i.e. those who are infected by external source. Most of them are young men aged 24–57; second, local residents in Shandong who are in close contact with the first group, i.e. those who are infected by internal spread. Places of spread include residential areas, supermarkets, hospitals, prisons, etc. People aged 30–60 are primarily infected groups with a gender balance. Based on the characteristics of susceptible population, the elderly with diabetes, coronary heart disease, cerebral infarction and other basic diseases are the high-risk population^8^.

## Spatio-temporal evolution patterns of the COVID-19 epidemic in Shandong province

### Temporal process

#### Daily evolution of the COVID-19 epidemic

**Figure 7:**
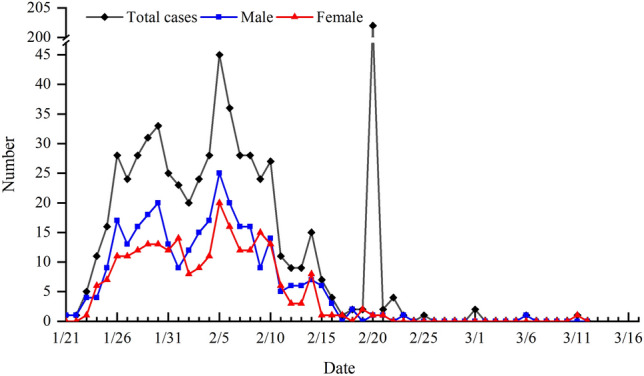
Change of the number of confirmed COVID-19 cases in Shandong province.

It can be seen from the number of daily confirmed cases in Shandong that February 5 was the turning point, showing a trend of “increase followed by decrease” (Fig. 7). Since the first confirmed case of external source was reported in Qingdao on January 21, the number of confirmed cases of this kind increased significantly from January 21 to 26, slowed down from January 27 to 30, and reached the third peak (33 cases) on January 30. The number of confirmed cases decreased significantly from January 30 to February 2; but increased to a subpeak (45 cases) from February 2 to 5, mainly due to close social contact around the period of the Spring Festival. From February 5 to 19, the number of confirmed cases decreased day by day, which was mainly related to scientific prevention and control measures in various regions. On February 20, 200 cases of cluster infection occurred in Rencheng prison which pushed the number of confirmed cases to the highest peak (202). From February 21 to March 12, there were no confirmed cases for many days, and the epidemic was almost controlled. In terms of gender, the first female case was confirmed on January 24, 3 days later than the first male case. The change of the number of confirmed male cases was consistent with the total number while the number of confirmed female cases increased slowly before February 5 because imported cases female were not many, albeit their number was basically consistent in the later period. In general, the number of confirmed cases per day was mainly male before February 5, but the cases were equal between male and female after February 5.

It’s worth noting that, the Rencheng Prison incident, happened on January 21, 2020, was a case of pandemic outbreak. It was spread by an individual who drove from Wuhan to Ji’ning, Shandong, infecting several police officers and criminals with the epidemic. The pandemic outbreak was considered an act of negligence, namely due to the prison polices, staffs and other police officers neglecting their duty, and thus causing epidemic prevention useless. In addition, the spread of the pandemic in Rencheng Prison was also the responsibility of both the Shandong Bureau of Justice and the individuals in charge of Prison Administrative Bureau, for neither of them was aware of the uniqueness, complexity or sensitivity of control the pandemic in prisons. Thus, individuals involved were charged with criminal responsibility. Closed Spaces, such as prisons and cruise ships, can easily lead to widespread infection. Relevant administrative departments should be on high alert to prevent similar accidents from happening again^22^.

#### Development of prevention and control policies

**Figure 8:**
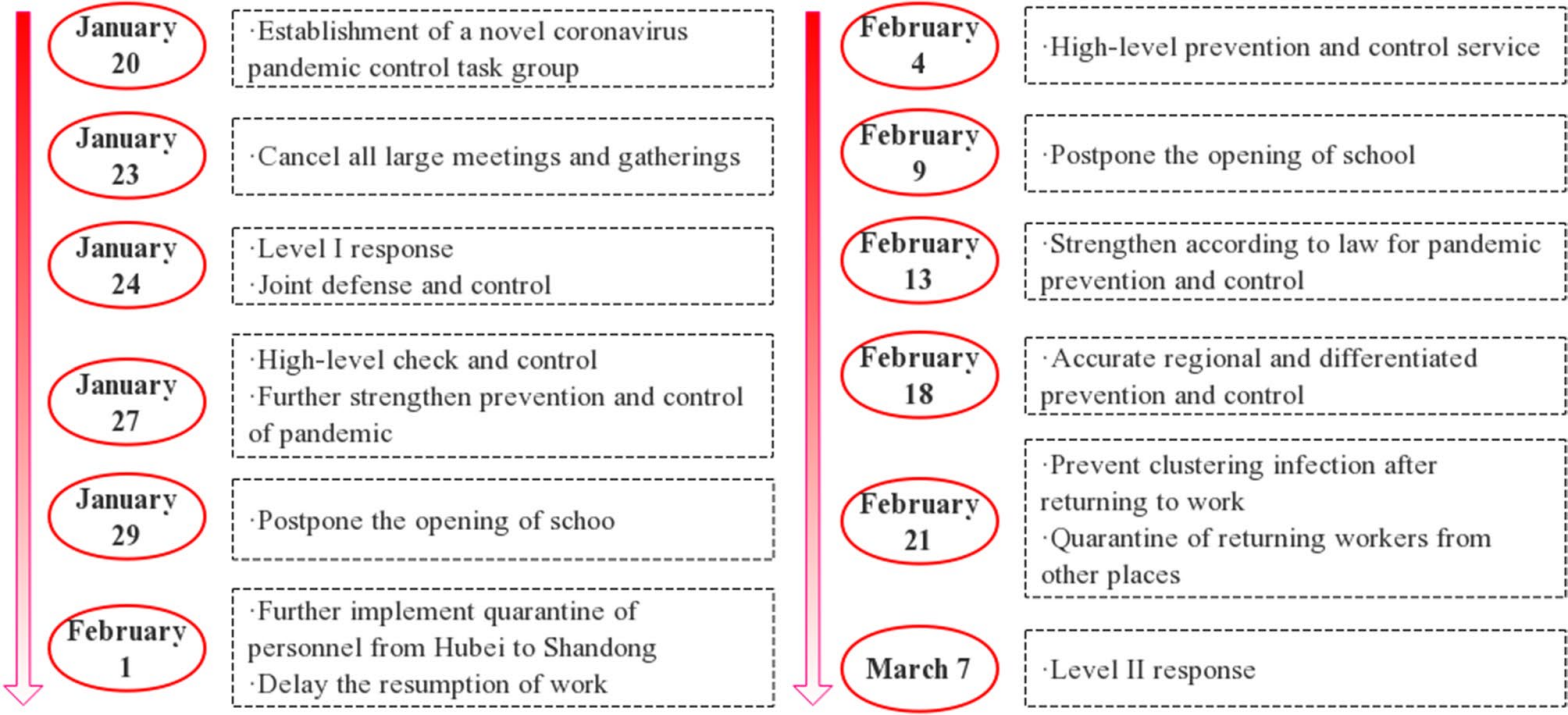
Evolution of COVID-19 epidemic prevention and control policies in Shandong province.

As shown in Fig. 8, on January 15, after the Center for Disease Control and Prevention (CDC) launched level I emergency response, Shandong province established epidemic prevention and control working group on January 20, and each city established a response command system as well. On January 23, the general office of the provincial Party committee called for the cancellation of large-scale meetings and gathering activities to prevent the spread of the epidemic. On the new year’s eve of January 24, the level I response to major public health emergencies was launched, and the epidemic prevention and control personnel canceled their holidays and fully participated in the prevention and control. On January 27, the government stressed the precise implementation of policies and decided to postpone the opening of universities, primary and secondary schools and kindergartens. On February 1, measures were implemented to quarantine visitors from Hubei province and reduce the number of people gathered to stop the spread of the epidemic. On February 13, epidemic prevention and control was strengthened in accordance with the law and elevated as a legal action. On February 18, differentiated and forced control was implemented to prevent the spread of the disease. On February 21, measures were taken to strictly prevent the collective infection of reopening of business, and the returning workers from other places were quarantined for observation. So far, the epidemic has been effectively controlled. On March 7, major public health emergency was adjusted to level II although the measures and the intensity of their implementation remained unchanged, and more targeted measures were taken to comprehensively promote epidemic prevention and control and economic and social development.

### Spatial pattern

#### Overall spatial distribution at city-level

**Figure 9:**
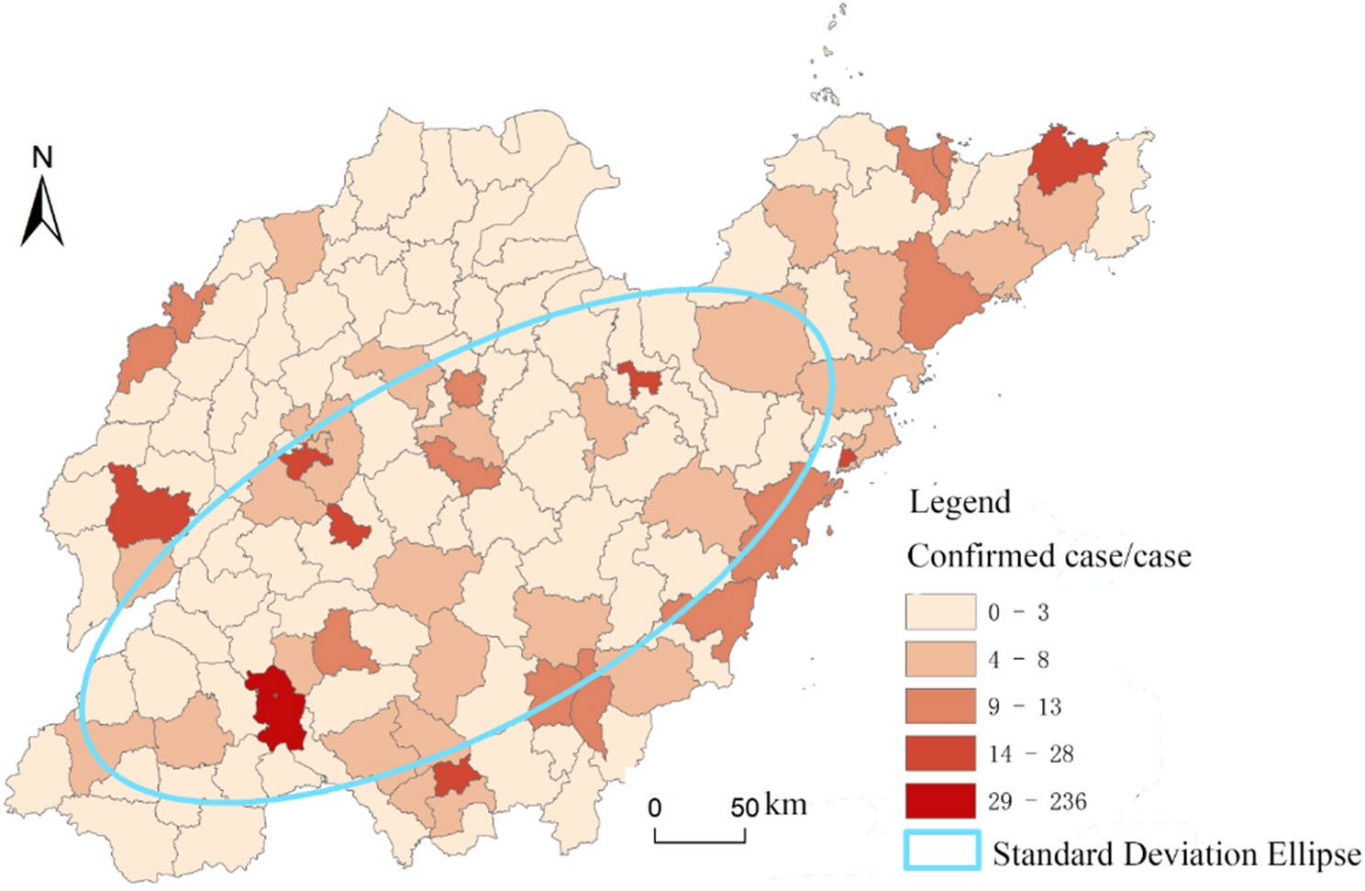
Spatial distribution of confirmed COVID-19 cases in Shandong province (this graph is produced by using ArcGIS10.3. http://www.esri.com; The base map is from China Standard Map Service. http://bzdt.ch.mnr.gov.cn/).

According to the spatial distribution map of confirmed cases of COVID-19 in Shandong province (Fig. 9), Ji’ning is the most seriously affected (260 cases) in terms of cities mainly due to the concentrated infections in Rencheng prison, followed by Qingdao, Linyi, Ji’nan, Yantai and Weifang, with 61, 49, 47, 47 and 44 cases respectively, but no confirmed cases were reported in Dongying. There was a significant correlation between the number of confirmed cases and the level of economic development, population size and the number of areas moved in (Table 2).

**Table 2:** Correlation index statistics of the number of confirmed COVID-19 patients at city-level.

	GDP/100 m yuan	Total population at end of year/10,000	No. of areas moved in
Pearson correlation coefficient	0.665**	0.716**	0.686**
Significance (double tail) index	0.005	0.002	0.003

**P < 0.01.

With regards to counties, the epidemic situation was divided into five levels, the first level is Rencheng district of Ji’ning; the second one includes six districts and counties such as the Zhongqu istrict of Ji’nan and Dongchangfu district of Liaocheng; the third level comprises 12 districts and counties, such as Wucheng county of Dezhou and Huangdao district of Qingdao; the fourth level includes 30 counties and districts, including Yanggu county of Liaocheng and Pingyi county of Linyi; the fifth level includes Dongming county of Heze, Linqu county of Weifang and other areas. It can be seen that the confirmed cases are mainly distributed in the southwest of province and Jiaodong Peninsula, going from southwest to northeast. Meanwhile, the cases in coastal areas are relatively dense. The distribution in all cities is generally decreasing from the main urban area to the surrounding areas and counties.

#### Spatial evolution process

**Figure 10:**
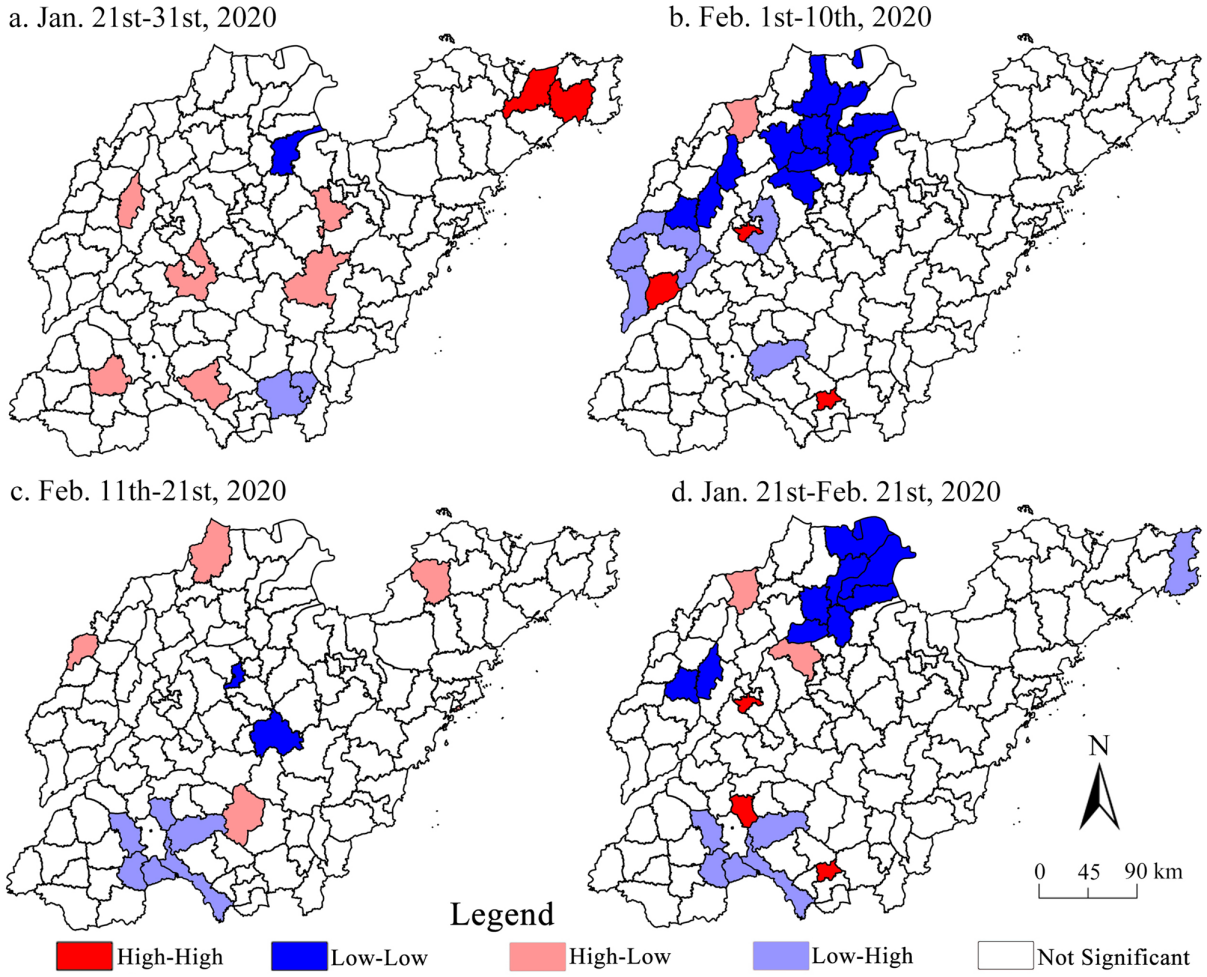
Hotspots and coldspots of confirmed COVID-19 cases at county-level in Shandong province (this graph is produced by using ArcGIS10.3. http://www.esri.com; The base map is from China Standard Map Service. http://bzdt.ch.mnr.gov.cn/).

According to the global Moran's *I* index of epidemic distribution in each time period calculated with the interval of 10 days (from February 22 to March 12, the number of confirmed cases too very small to be discussed here) (Table 3), it is found that since the outbreak of the epidemic in Shandong, the county-wide Moran’s *I* index is negative, and the *Z* value is greater than − 1.65, indicating that the epidemic in Shandong presents the clustering characteristics of “high–low” and “low–high” pattern, that is, the high (low) incidence counties and surrounding low (high) incidence counties are huddled together (Fig. 10). In most of the districts (counties) of Shandong, the clustering characteristics of the diagnosed groups are not significant, although it became especially obvious in the third stage. The characteristics of full-time clustering, mainly referring to “high–high” type, include the city centers of Ji’nan, Ji’ning and Linyi, because the terrain is relatively flat, the temperature is suitable, and the precipitation is relatively rich, which is conducive to the development of crops. Good natural factors lead to the dense population in such areas^13^, especially in the downtown area, the “low–high” type are mainly concentrated in the surrounding districts and counties of Ji’ning city, although the population here is relatively dense, it is separated by Weishan Lake (the largest fresh water lake in Shandong province). The traffic is relatively underdeveloped, which is easy to prevent and control the epidemic (traffic control). Secondly, large-scale infection event (central area) occurs in Rencheng prison, which leads to the “low–high” clustering characteristics of surrounding districts and counties, the “low–low” type are mainly concentrated in Binzhou and Dongying, located in the Yellow River Delta. Although it is located in the plain, it is widely distributed due to strong coastal erosion, which is not conducive to agricultural production, resulting in sparse population distribution. In general, after Shandong launched the level I response to major public health emergencies, effective regional prevention and control measures weakened the correlation of regional of spread of epidemic.

**Table 3:** Global Moran's I statistics of the COVID-19 epidemic in Shandong province.

Date	Moran’s *I*	*Z*	*P*	Date	Moran’s *I*	*Z*	*P*
January 21st–31st	0.083***	1.9269	0.034	February 1st–10st	0.017	0.4829*	0.269
February 11st–21st	− 0.031**	− 1.2521	0.082	January 21st to February 21st	− 0.023	− 1.3632*	0.168

***P ≤ 0.05; **P ≤ 0.1; *P ≤ 0.5.

### Evolution of migration network

**Figure 11:**
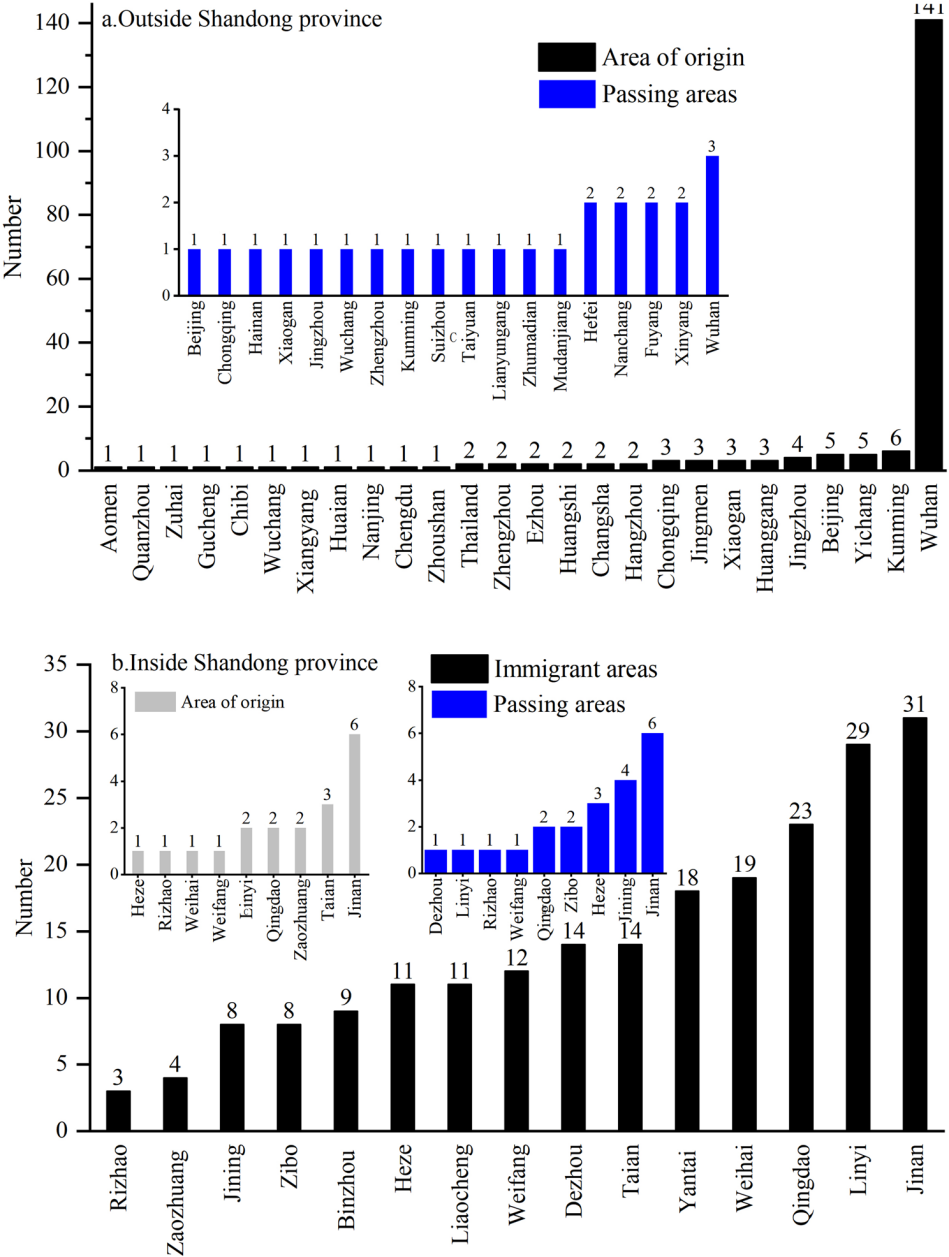
Distribution of migration track points of confirmed COVID-19 cases in Shandong province.

**Figure 12:**
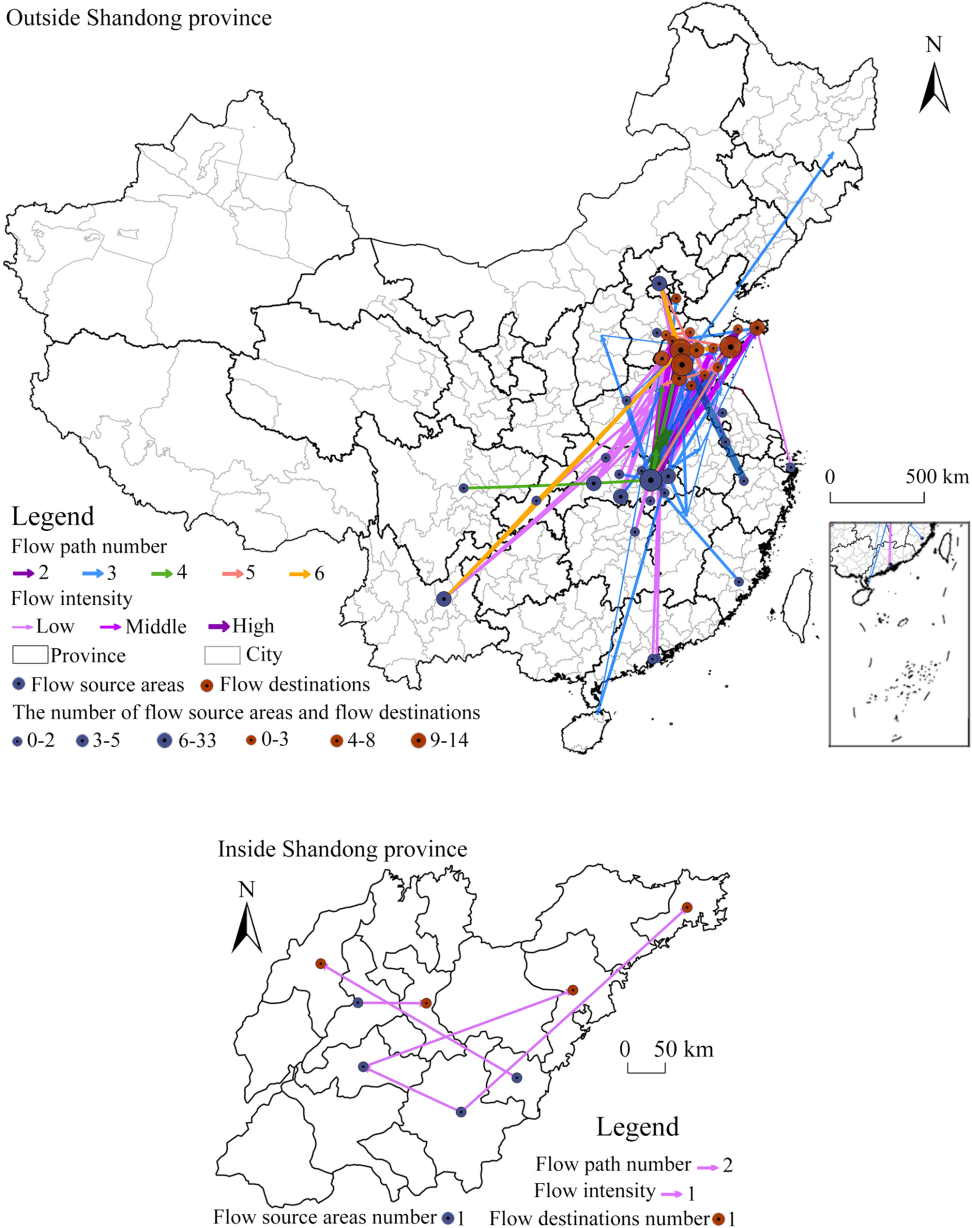
Spatial flow paths of confirmed COVID-19 cases in Shandong province (this graph is produced by using ArcGIS10.3. http://www.esri.com; The base map is from China Standard Map Service. http://bzdt.ch.mnr.gov.cn/).

Shandong is a province far away from the epicenter of the epidemic but with a high incidence of cases in China. According to the information of spatial migration track of confirmed cases, the distribution map of migration track points (Fig. 11) and spatial flow path map (Fig. 12) are drawn, and the characteristics of migration track points and spatial flow direction are analyzed (excluding three international roads of “Thailand → Qingdao”, “Ji’nan → Thailand → Ji’nan” and “Italy → Beijing → Qingdao → Pingdu”).

#### Track point distribution

The places for emigration are mainly outside the province, distributed in 13 provinces and cities of the country as well as Thailand and Italy, of which Hubei is the main emigrated province, and Wuhan (141 cases) the main emigrated region. It is followed by Kunming, Chongqing and Beijing with 5, 5 and 3 cases respectively, because they are the hot cities of migration during the Spring Festival owing to economic, political and cultural factors. The migrated places within the province involve 9 prefecture-level cities: Ji’nan (6 times), the largest number, followed by Tai’an (3 times).

There is no significant difference in the number of passing areas inside and outside the province. They are distributed in 18 prefecture-level cities in 10 provinces and cities across the country. Wuhan, Suizhou, Jingzhou, Xiaogan and other cities in Hubei are the main passing places outside the province, followed by regions between Shandong and Hubei, such as Xinyang in Henan (the number of confirmed cases in Henan ranks the third in China), Fuyang in Anhui (the number of confirmed cases in Anhui ranks the sixth in China). In Shandong, passing areas are more concentrated in 9 cities with Ji’nan and Ji’ning with most people because they are the two railway transportation hubs in the province.

The moving-in places are located in 15 cities in Shandong, with Ji’nan, Linyi, Qingdao, Weihai, and Yantai as the primary sites, followed by Tai’an, Dezhou, Weifang, Liaocheng, and Heze. The number of areas moved in is significantly related to the level of economic development, population size and the number of confirmed cases (Table 4).

**Table 4:** Correlation index statistics of the number of destinations.

	GDP/100 m yuan	Total population at end of year/10,000	Number of confirmed cases
Pearson correlation coefficient	0.599**	0.619**	0.686**
Significance (double tail) index	0.014	0.011	0.003

**P < 0.01.

#### Spatial flow network

The spatial flow path of confirmed cases (Fig. 12) is dominated by cross-province flow, having the characteristics of “Hubei → Shandong” intensive flow, and “other places → Shandong” dispersive flow, with an overall trend of “southwest-northeast” mobility. The largest number of track point is 6 with a total of 174 direct flow routes. The “outside → inside the province” flow includes 169 routes, such as “Wuhan → Ji’nan”, “Beijin → Dezhou” while the “within province” flow includes 5 routes, such as “Tai’an → Qingdao” and “Rizhao → Dezhou”. The main reasons for direct flow are living, tourism, work and study outside the province. The remaining 40 flow paths include a single movement in one or many cities in Hubei or a multi-point mixed mobility in other cities outside Hubei. The 3-point travel mainly involves visiting relatives and traveling outside the province, while the 4-, 5- and 6-point travel mainly involves business trips to multiple places.

## Prevention and control mechanism of COVID-19 epidemic in Shandong province

**Figure 13:**
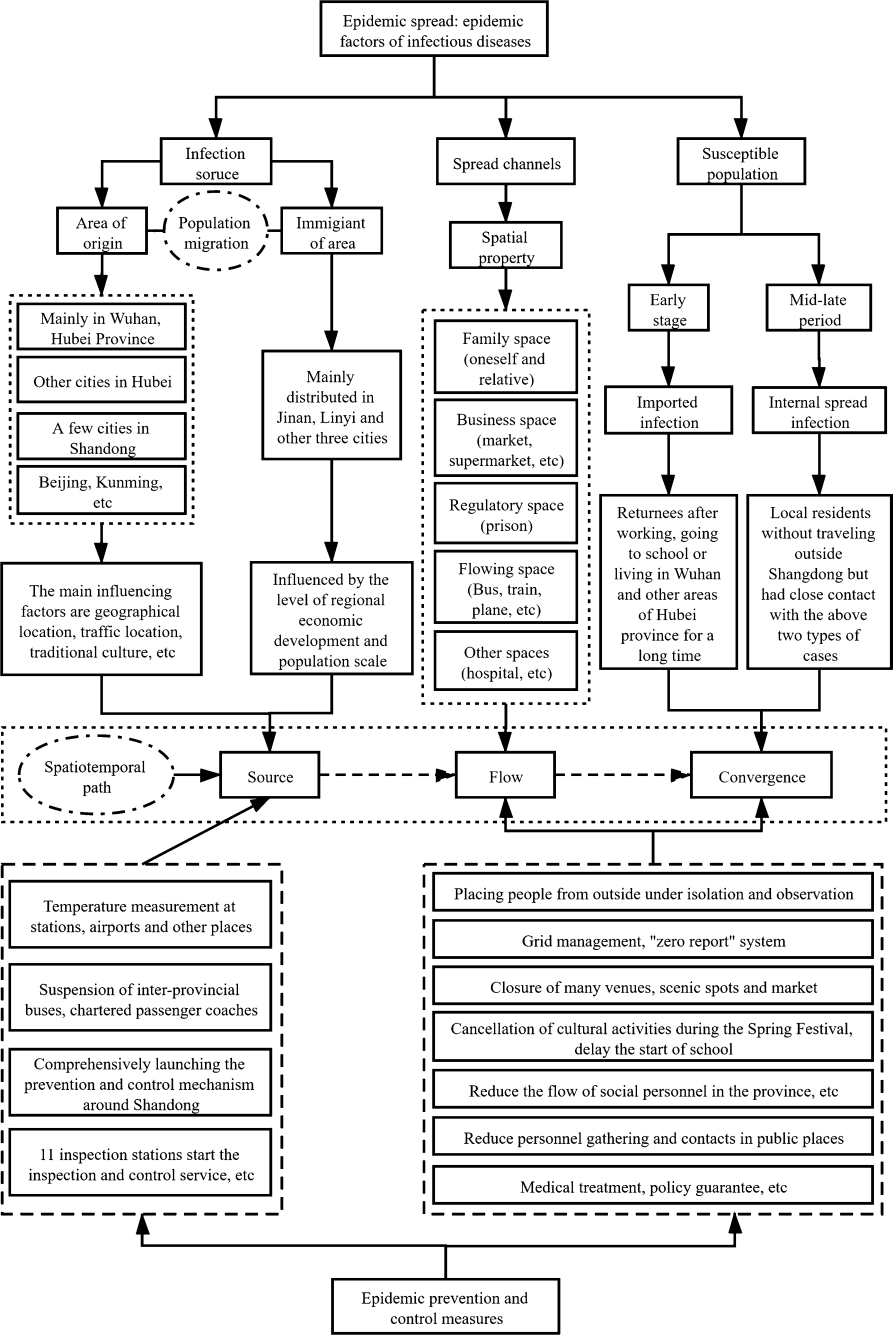
Spatio-temporal evolution and control mechanism of COVID-19 epidemic in Shandong province.

According to the theory of infectious diseases and the epidemic spread characteristics of “source-flow-convergence”, the spread evolution of epidemic and comprehensive mechanism of prevention and control in Shandong province is explored (Fig. 13). In Shandong, a province far from the epicenter of the epidemic but with a high incidence of cases in China, those who were infected by migration to other regions are regarded as the “source”; the transmission space that provides necessary space for the spreading path is regarded as the “flow”; the general susceptible population is regarded as the “convergence”. Therefore, external prevention and control measures, such as suspending inter-provincial buses, chartered passenger coaches, comprehensively launching the prevention and control mechanism around Shandong, were taken to effectively control the “source”; internal prevention and control measures, such as placing people from outside under isolation and observation, canceling recreational activities, delaying the start of school, reducing personnel gathering and contacts, were taken to block the spread channels. Measures and policies were also taken to protect vulnerable people and provide medical treatment by jointly forming a systematic prevention and control mechanism^23^. In general, the effective prevention and control of the epidemic was due to the advantages of the social system. All departments in the province reacted quickly and cooperated effectively to win the key battle of the prevention and control of the epidemic.

## Conclusion and discussion

### Conclusion

COVID-19 has caused great harm to the society, and has become a serious concern of the people. From the geographical perspective, this paper uses text analysis, mathematical statistics and spatial analysis to analyze the epidemiological characteristics of confirmed cases and the temporal and spatial evolution characteristics of the epidemic in Shandong province, which is a province far from Wuhan but with a high incidence of cases in China. The main conclusions are as follows:

1. Epidemiological characteristics: the incidence rate of COVID-19 in Shandong is 0.76/100,000 and male cases are slightly more than female cases. The male population with spatial migration is the main carriers of the epidemic. The age is mainly concentrated in 30–60 years old. The main group with spatial mobility is middle-aged and young people, and the local residents are mainly middle-aged and elderly. The main routes of transmission are internal spread followed by external source and mixed transitional spread. The main types of spread are the cross-province staying, major events and family gathering, and the most susceptible groups are the migration groups and their close contacts.
2. Spatio-temporal evolution pattern: in terms of time, the number of daily confirmed cases decreased first and then increased on February 5, 2020. As of February 21, the epidemic was basically controlled. The prevention and control policies directly affected the spread of the epidemic, which lasted 42 days, so the level I response to major public health emergencies was adjusted to level II. Spatially, citywide distribution of confirmed cases is unbalanced: the epidemic decreased from the center of the city to the surrounding districts and counties, which is negatively correlated, showing the “high–low” and “low–high” clustering characteristics.
3. Spatial flow network: affected by the geographical location, traffic accessibility of Wuhan and Chinese traditional culture, Wuhan and other cities in Hubei province are the main places for people to move out and pass through; Ji’nan, Linyi, Qingdao, Weihai and Yantai are the main places to move in; and the number of places to move in is significantly related to the level of economic development and population size, and highly related to the number of confirmed cases. The spatial flow direction is mainly inter-provincial, with most mobility goes from Hubei to Shandong and scattered mobility from other regions to Shandong. The primary flow is the point-to-point migration, going from “Southwest-northeast”.
4. Based on the epidemic theory of infectious diseases and the spreading characteristics of “source-flow-convergence” of the epidemic, the epidemic evolution and the comprehensive mechanism of prevention and control in Shandong is explored. Through the implementation of a number of effective measures aimed at controlling the “source” and blocking the spread path and protecting the vulnerable population, the epidemic can be effectively controlled.

### Discussion

The epidemic in Shandong has been controlled through the cooperation of various departments and all sectors of society. In the future, it is still necessary to strengthen the follow-up investigation of medical records of discharged patients and the monitoring of the floating population, as well as the screening management of the returned personnel from other places and the local residents going to other provinces, and emphasize the importance of self-protection. In the further prevention and control of the epidemic, we should promote the economic and social development as a whole, prevent the rebound caused by the resumption of work and returning to school, strengthen the public monitoring of the epidemic, actively carry out patriotic education and publicity about popular science, and guide and tackle other social problems caused by the epidemic. In addition, although the epidemic peak of the current round in China has passed, it is still in spreading wildly in many countries. At present, imported cases have occurred in Shandong province. We should be alert to the recurrence of the epidemic caused by imported cases.

This paper focuses on revealing the spatio-temporal evolution, characteristics of migration pattern evolution and prevention and control mechanism of the COVID-19 in Shandong province, which is an active attempt from the perspective of a remote region far from the epicenter of the epidemic yet with a high incidence of cases. In the future, further work will be carried out from the following aspects: (1) carry out epidemic research focusing on the high incidence cities, interpret the evolution of the epidemic and prevention and control mechanism microscopically and make a comparative analysis. In view of the infection incident in Rencheng prison, try to analyze the causes of the outbreak evolution and explore the prevention and control mechanism of regulatory space. (2) According to the characteristics of domestic and foreign epidemic development and considering the actual situation and demand of returning to work and school, a typical scenario will be constructed and a targeted prevention and control plan will be worked out. (3) Continue to follow up and pay attention to the worldwide spread of COVID-19 and imported cases at home and abroad, explore the global spread of COVID-19 and estimate the risk of imported cases.

This paper has the following limitations. First, the analysis based on spatial data lacks attention to the perspective of individuals and groups. We will focus on the differences between different characteristic groups in different regions in future work. Second, in current period, the relationship between prevention policy and the development of the epidemic deserves further discussion.

## Data Availability

All the data in this paper are from the government public website.
